# Increase of Intracellular Zinc Levels Rather Than Zinc Influx Inhibits Interleukin‐2 Production in Zinc Supplemented Jurkat Cells

**DOI:** 10.1002/cbf.70098

**Published:** 2025-07-02

**Authors:** Christian M. Sobernig, Henrike J. Fischer, Lothar Rink, Jana Jakobs

**Affiliations:** ^1^ Institute of Immunology Medical Faculty RWTH Aachen University Aachen

**Keywords:** calcimycin, IL‐2, mitogens, T cells, zinc

## Abstract

The essential trace element zinc is a well‐known modulator of T cell activation. There have been contradictory findings for the impact of zinc supplementation on T cell activation. In our study, we aimed to analyze IL‐2 production in Jurkat T cells during zinc supplementation in response to different stimuli. We found that zinc strongly suppresses IL‐2 production in Jurkat cells stimulated with phorbol 12‐myristate 13‐acetate (PMA)/calcimycin or phytohemagglutinin (PHA)/calcimycin. In contrast, zinc had no impact on IL‐2 production after PHA stimulation alone, suggesting the inhibitory zinc‐effect was linked to high calcium influx. To distinguish if the observed IL‐2 suppression is due to either potential competing effects of zinc influx or simple elevation of intracellular zinc levels, we pretreated the Jurkat cells with the zinc ionophore pyrithione for an increase of intracellular zinc before the stimulation. It was sufficient to suppress IL‐2 expression even when the cells were not further supplemented with zinc during stimulation. We propose that zinc's inhibitory effects on phosphatases stabilize the phosphorylated NFAT and thus block IL‐2 expression. Our findings underline the importance of a balanced zinc status for proper immune functions and suggest a supporting effect of zinc during immunosuppressive treatments.

## Summary

- Zinc is a critical trace element involved in immune regulation. Our study shows that increased intracellular zinc levels suppress IL‐2 production in Jurkat T cells in a stimulus‐dependent manner.
- This effect is linked to calcium‐mediated signaling and may result from zinc‐induced inhibition of phosphatases, leading to persistent NFAT phosphorylation and thus to reduced transcription of IL‐2.
- These findings provide new insights into the molecular mechanisms by which zinc modulates T cell function and suggest that intracellular zinc homeostasis is a key factor in immune regulation, with potential implications for immunosuppressive strategies.

**Abbreviations**
Cal: calcimycin
CN: calcineurin
CRAC: calcium release‐activated Ca^2+^ channels
DAG: diacylglycerol
IP_3_
: inositol trisphosphate
NFAT: nuclear factor of activated T cells
NFκB: nuclear factor k‐light‐chain‐enhancer of activated B cells
PHA: phytohemagglutinin
PLC: phospholipase C
PMA: phorbol 12‐myristate 13‐acetate
TCR: T cell receptor
Th: T helper cell
Treg: regulatory T cell

## 1 Introduction

Over the last decades it has been recognized that the essential trace element zinc plays a substantial role in cellular immunity [1]. It was demonstrated that zinc has various effects on T cells [2]. Zinc deficiency causes impaired development and alters polarization into effector T cells [3, 4]. Different working groups reported that zinc supplementation decreases numbers of T helper cells (Th)2 and Th17 and enhances the proportion of Th1 and regulatory T cells (Treg cells) which may suppress excessive immune responses [5, 6]. Zinc also exhibits a strong influence on signaling pathways regulated by Ca^2+^, that is, its inhibitory effect on Ca^2+^/Calmodulin‐Stimulated Protein Kinase II (CaMPK‐II) [7].

An inhibiting effect of zinc on the phosphatase calcineurin (CN), activated by mitogens has been discovered in vitro [8]. The CN signaling pathway plays a critical role in T lymphocyte activation, starting with engagement of the T cell receptor (TCR). Subsequently, phosphorylation of phospholipase C‐γ (PLC‐γ) results in hydrolysis of phosphatidylinositol 4,5‐bisphosphate to produce the second messengers diacylglycerol (DAG) and inositol trisphosphate (IP_3_). IP_3_ triggers the release of Ca^2+^ from ER, promoting entry of extracellular Ca^2+^ into cells through calcium release‐activated Ca^2+^ (CRAC) channels. Ca^2+^‐bound calmodulin then activates the phosphatase CN, promoting IL‐2 gene transcription via the nuclear factor of activated T‐cells (NFAT) [9, 10, 11, 12]. IL‐2 is a cytokine that has a decisive impact on proper immune functions by causing proliferation and differentiation of activated T cells, regulatory T cells, and other cells of the innate and adaptive immune system [13, 14]. It is long‐established that IL‐2 production is stimulated by mitogens like phorbol 12‐myristate 13‐acetate (PMA) in combination with the ionophore calcimycin (Cal) in Jurkat T cells. Another mitogen is the lectin phytohemagglutinin (PHA) [15, 16]. These stimulators have different mechanisms of action: PMA directly activates the protein kinase C (PKC), which can activate numerous other pathways and kinases, such as nuclear factor κ‐light‐chain‐enhancer of activated B cells (NFκB) [17, 18]. It has been described that the combination of PMA with Cal is required for the stimulating effect [19]. Cal has been discovered to increase the concentration of intracellular Ca^2+^ via calcium influx [20, 21]. PHA is a potent stimulator of the calcineurin/NFAT pathway activation; however, it is not fully understood which signaling molecules are affected. Previous experiments showed that PHA binds directly to the TCR [22], while others found a direct effect on NFAT [23].

It was described that zinc deficiency in Jurkat T cells led to decreased IL‐2 expression [24]. Prasad et al. also described an increased IL‐2 expression after zinc supplementation (ZS) in zinc‐deficient HUT‐78 cells caused by NFκB activation [25]. In line with this, in zinc‐deficient murine EL‐4 6.1 T cells, a supplementation with zinc upregulated IL‐1β‐induced IL‐2 production efficaciously [26]. In contrast, zinc was shown to inhibit mitogen‐induced IL‐2 expression [27]. Thus, we aimed to answer the questions: Does zinc have a supporting or suppressing effect on IL‐2 expression in Jurkat T cells? And is this effect depending on the stimulating agent?

To this end, we used Jurkat T cells cultivated under zinc adequate conditions to investigate the effect of zinc supplementation on the CN signaling pathway, activated by PMA/Cal or PHA. We could show that increased intracellular zinc levels lead to the suppression of IL‐2 production in Jurkat cells accompanied by reduced dephosphorylation of NFAT independently of the increased influx of extracellular zinc during stimulation and rather based on elevated intracellular zinc levels. One possible explanation could be the previously described ability of zinc to block calcium channels [28]. Our findings suggest that zinc does not have a strong impact on intracellular calcium levels. It opens a new perspective on zinc, which uses a still unknown signaling pathway to inhibit IL‐2.

## 2 Methods and Materials

### 2.1 Cell Culture and Experimental Setup

The human T cell line Jurkat was cultivated at 37°C in a humidified 5% CO_2_ atmosphere in RPMI 1640 media containing 10% FCS (Bio&SELL, Germany), 2mM l‐glutamine, 100 U/mL potassium penicillin, 100 U/mL streptomycin sulfate, 1% non‐essential amino acids and 100 mM sodium pyruvate (all from Sigma‐Aldrich, Germany).

**Figure 1:**
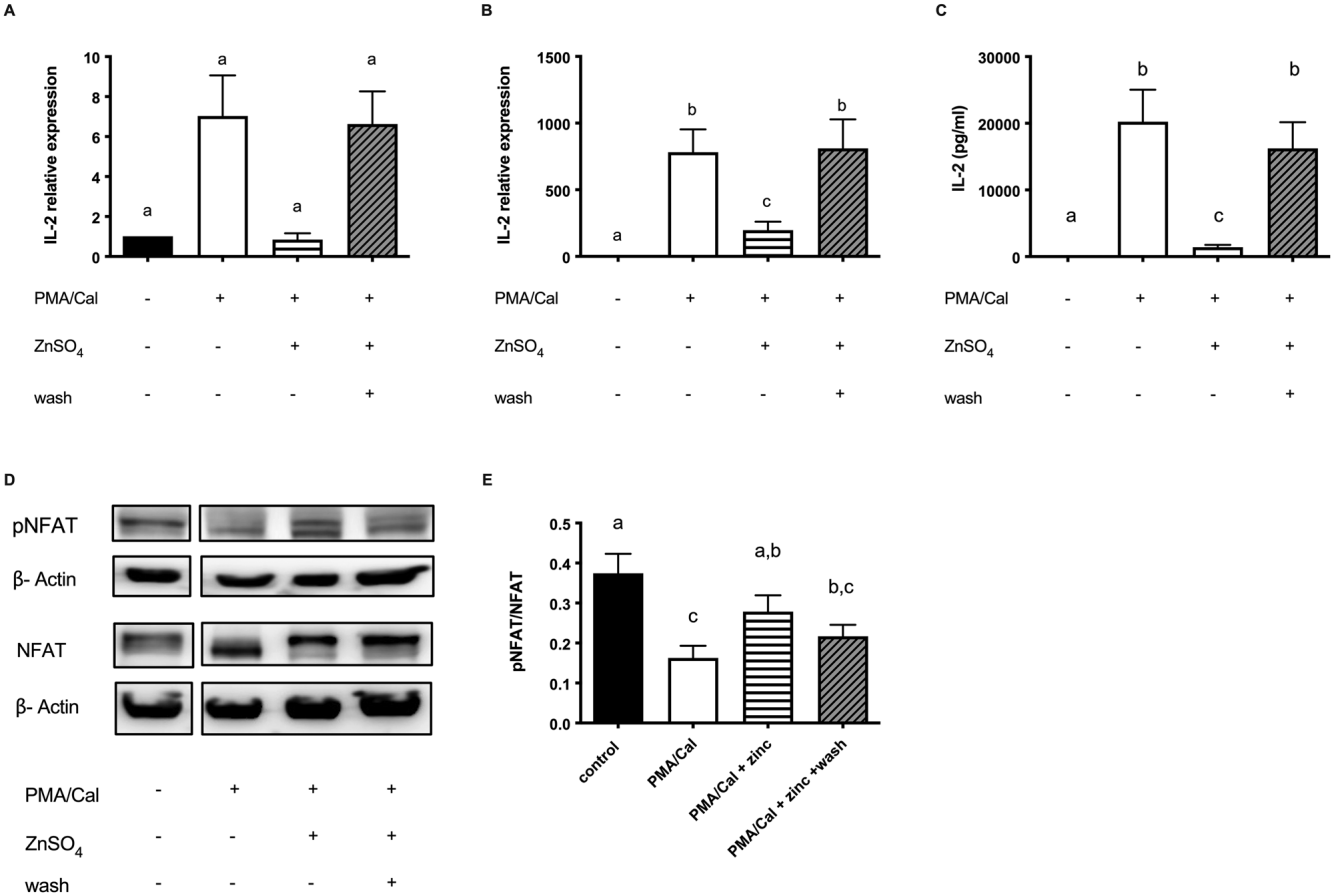
Zinc inhibits the PMA/Cal activated NFAT signaling pathway. Jurkat cells were stimulated as described in the experimental section. (A) qPCR was performed to quantify IL‐2 mRNA after 3 h (*n* = 6) and (B) 6 h (*n* = 11). (C) IL‐2 secretion after 24 h, measured by ELISA (*n* = 11). (D) One representative western blot after 30 min is shown. The control band belongs to the same membrane but is excised and shown individually, as the samples stimulated with PHA are not shown. (E) pNFAT and NFAT (*n* = 9) were normalized to β‐actin on their respective membrane and their ratio was calculated. The controls shown in A, B, C, and E are also used in Figure 3A, B, C, and E, respectively. Results show mean values + SEM. Data were analyzed by repeated‐measures ANOVA and Tukey post hoc test (A–C, E). Significantly different means do not share the same letters.

**Figure 2:**
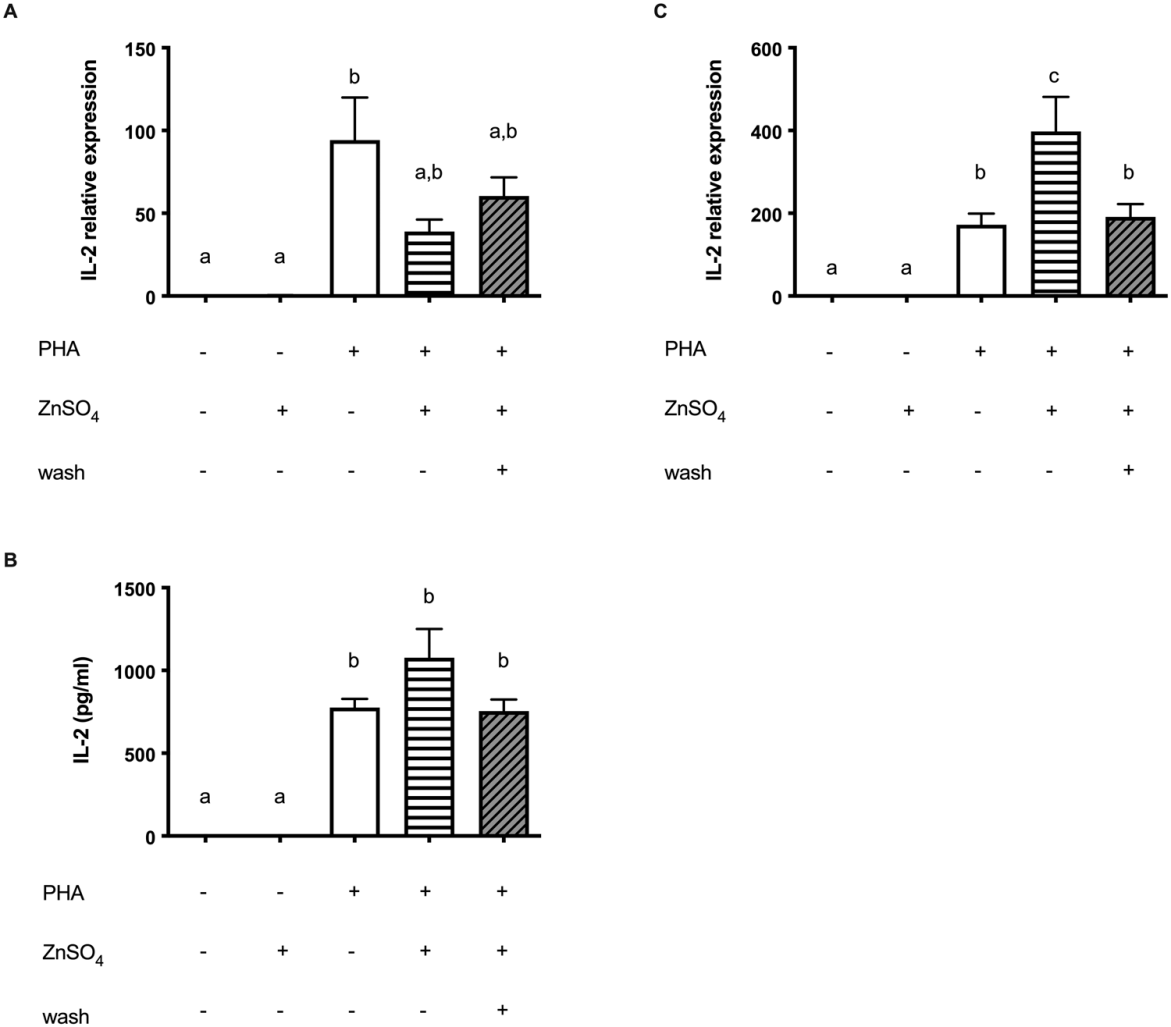
Zinc has no inhibiting effect on PHA stimulated IL‐2 expression. Jurkat cells were stimulated as described in the experimental section. (A) qPCR was performed to quantify IL‐2 mRNA after 3 h (*n* = 7) and (B) 6 h (*n* = 15). (C) IL‐2 secretion after 24 h measured by ELISA (*n* = 10). Results show mean values + SEM. Data were analyzed by repeated‐measures ANOVA Friedman test and Dunn's post hoc test (A) and Tukey post hoc test (B and C). Significantly different means do not share the same letters.

**Figure 3:**
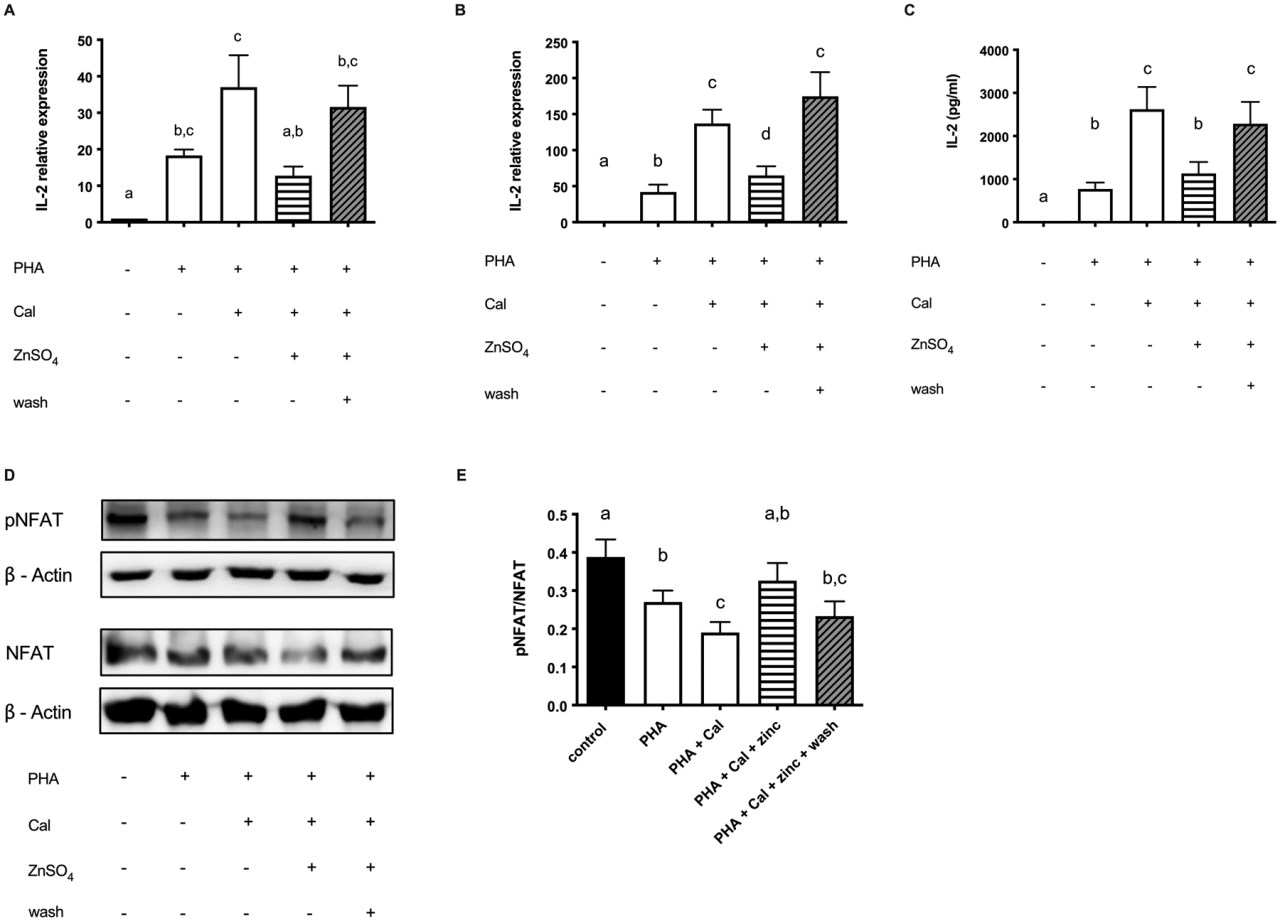
Zinc inhibits PHA/Cal stimulated NFAT signaling pathway. Jurkat cells were stimulated as described in the experimental section. (A) qPCR was performed to quantify IL‐2 mRNA after 3 h (*n* = 8) and (B) 6 h (*n* = 9). (C) IL‐2 secretion after 24 h, measured by ELISA (*n* = 8). (D) One representative experiment after 30 min is shown. (E) pNFAT and NFAT (*n* = 10) were normalized to β‐actin on their respective membrane and their ratio was calculated. The controls shown in A–C, E are also used in Figure 1A–C, E, respectively. Results show mean values + SEM. Data were analyzed by repeated‐measures ANOVA Friedman test and Dunn's post hoc test (A) and Tukey post hoc test (B–C, E). Significantly different means do not share the same letters.

In all our experimental setups, 1 × 10^6^ Jurkat cells in a concentration of 5 × 10^5^ cells/mL were used. For Figures 1, 2, 3, cells were either left untreated or were preincubated with 50 µM ZnSO_4_ (zinc sulfate, Sigma‐Aldrich) for 15 min. After preincubation, all samples were centrifuged and resuspended in their own medium, except for one, where zinc‐supplemented medium was exchanged with zinc‐adequate medium, indicated as “wash” throughout the manuscript. Zinc adequate medium does not contain additional zinc supplementation. Cells were then stimulated with either 2.5 µg/mL PHA (Biochrom, Germany) optionally combined with 1 µM calcimycin (Cal) A23187 (Sigma Aldrich), or 10 ng/mL PMA (Sigma Aldrich) compulsory combined with 1 µM Cal A23187 (Sigma Aldrich), or left untreated as control for 30 min, 3, 6, and 24 h.

**Figure 4:**
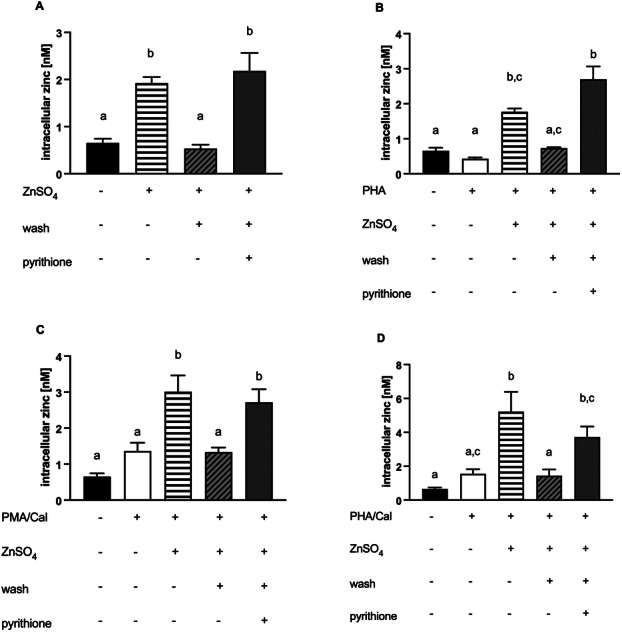
Pyrithione leads to a strong and persistent increased intracellular zinc amount. Jurkat cells were stimulated as described in the experimental section. After 3 h, labile zinc was measured with FluoZin‐3AM. (A) Cells after preincubation without stimulation (*n* = 5) or with (B) PHA (*n* = 10), (C) PMA/Cal (*n* = 7), or (D) PHA/Cal (*n* = 7) stimulation. Results show mean values + SEM. Data were analyzed by repeated‐measures ANOVA and Tukey post hoc test (A), (C) and (D) and Kruskal–Wallis test and Dunn's post hoc test (B). Significantly different means do not share the same letters.

**Figure 5:**
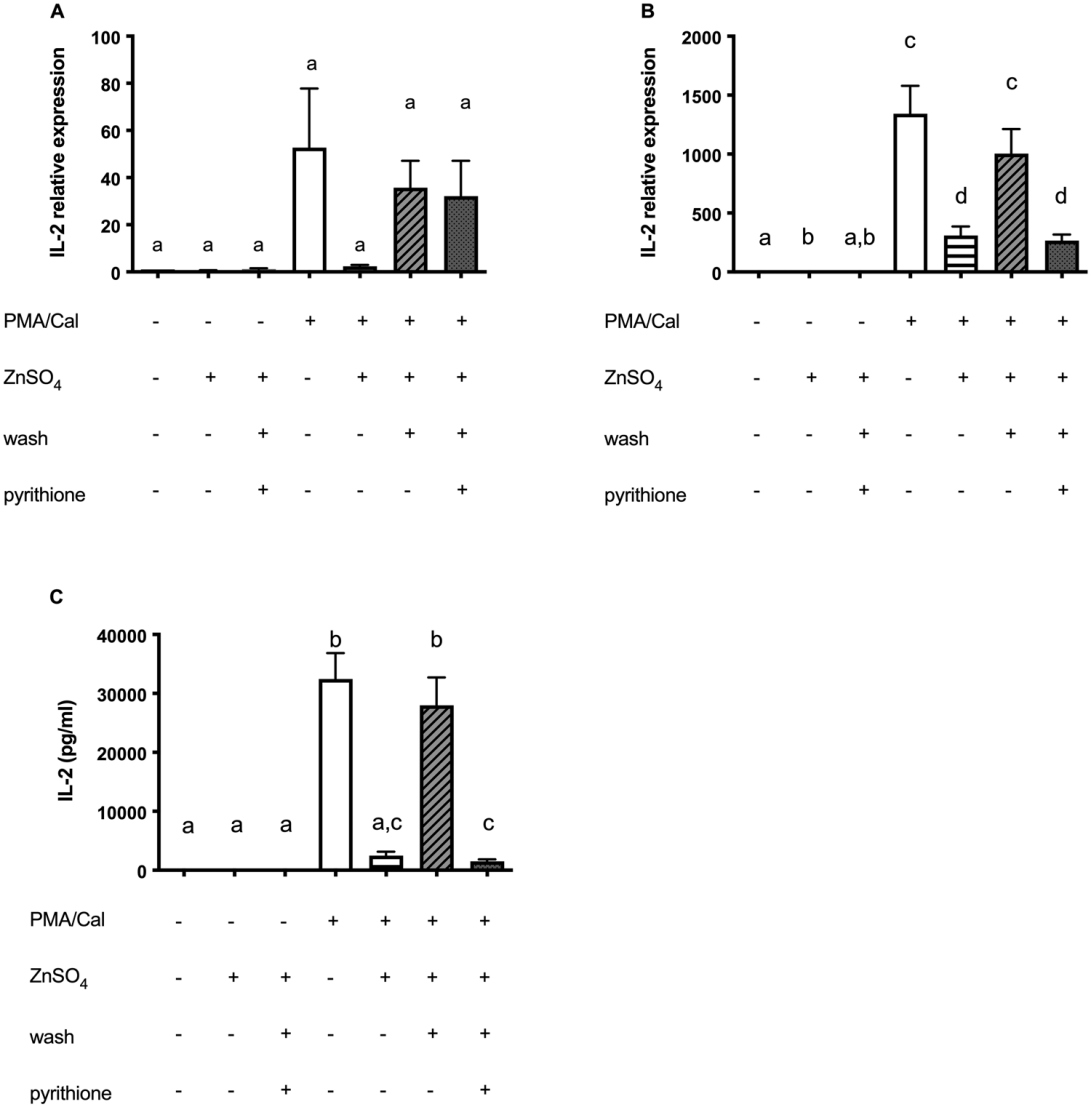
PMA/Cal‐induced IL‐2 production. Jurkat cells were stimulated as described in the experimental section. (A) qPCR was performed to quantify IL‐2 mRNA after 3 h (*n* = 4) and (B) 6 h (*n* = 14). (C) IL‐2 (*n* = 9) secretion after 24 h, measured by ELISA. The controls shown in A–C are also used in Figure 6A–C, respectively. Results show mean values + SEM of densitometric quantifications and measured results. Data were analyzed by repeated‐measures ANOVA Friedman test and Dunn's post hoc test (A) and Tukey post hoc test (B–C). Significantly different means do not share the same letters.

**Figure 6:**
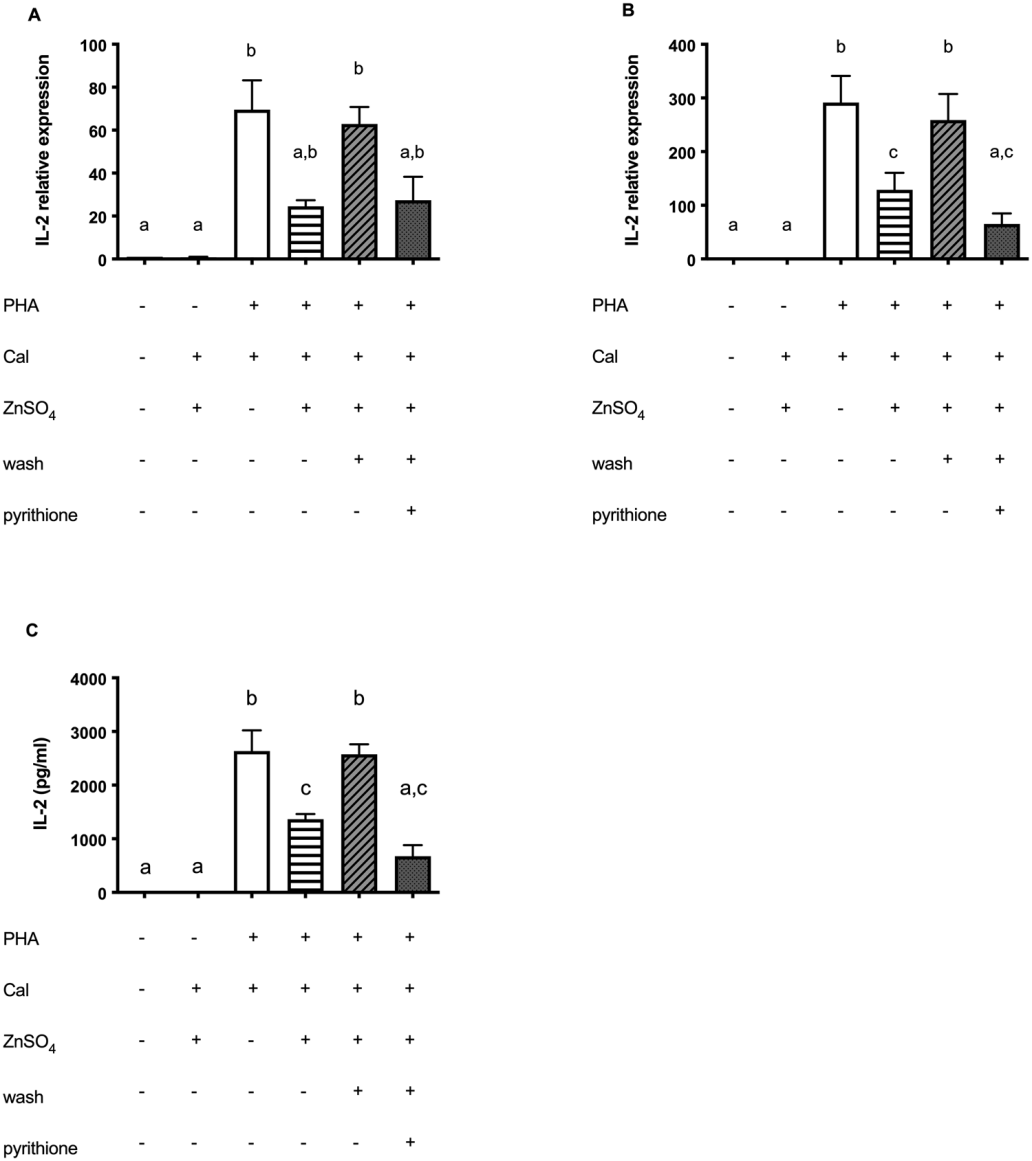
PHA/Cal‐induced IL‐2 production. Jurkat cells were stimulated as described in the experimental section. (A) qPCR was performed to quantify IL‐2 mRNA after 3 h (*n* = 8) and (B) 6 h (*n* = 11). (C) IL‐2 secretion after 24 h, measured by ELISA (*n* = 10). The controls shown in A–C are also used in Figure 5A–C, respectively. Results show mean values + SEM. Data were analyzed by repeated‐measures ANOVA Friedman test and Dunn's post hoc test (A) and Tukey post hoc test (B–C). Significantly different means do not share the same letters.

**Figure 7:**
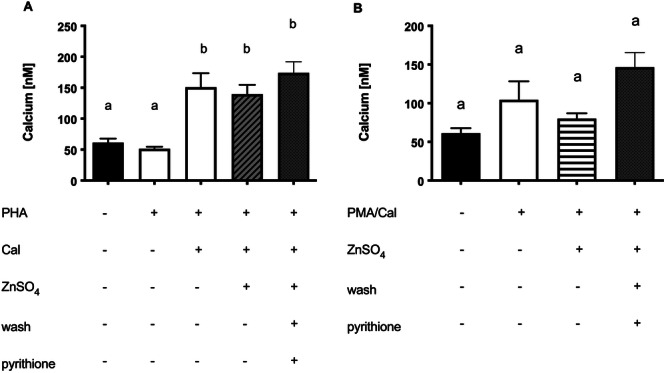
Zinc has no impact on calcimycin‐stimulated calcium influx. Jurkat cells were stimulated as described in the experimental section. Calcium was measured with Fluo‐4. (A) PHA and/or Cal (*n* = 5), or (B) PMA/Cal (*n* = 5) was used for stimulation. Same control was used in (A) and (B). Results show mean values + SEM. Data were analyzed by repeated‐measures ANOVA and Tukey post hoc test. Significantly different means do not share the same letters.

For Figures 4, 5, 6, 7, we added 1 µM pyrithione (Sigma Aldrich) to maximize the intracellular zinc levels during 15 min preincubation. Jurkat cells were treated as described above, whereby the pyrithione‐containing sample was resuspended in fresh medium like the “wash” sample after preincubation.

### 2.2 Quantitative Real‐Time Polymerase Chain Reaction (qPCR)

RNA isolation and cDNA synthesis were performed according to the manufacturer's instructions using the extractme total RNA kit (Blirt S.A., Poland) and the qScript cDNA synthesis kit (QuantaBioscience, Germany), respectively. Quantitative analysis was performed using the fluorescent SYBR green reagent on a QuantStudio 3 cycler (Applied Biosystems, Germany) for IL‐2 (95°C for 10 min, followed by 40 cycles at 95°C for 15 s and 58°C for 30 s) and GAPDH as housekeeping gene (95°C for 15 min, followed by 40 cycles of 95°C for 30 s and 60°C for 1 min). The following primers were used: IL‐2 (forward primer: TCCTGTCTTGCATTGCACTAAG, reverse primer: CATCCTGGTGAGTTTGGGATTC) GAPDH (forward primer: GAAGGTGAAGGTCGGAGTC, reverse primer: GAAGATGGTGATGGGATTTC). All samples were run in duplicates. Gene expression was quantified by use of the $2^{‐∆∆C_{T}}$ method as described before [29].

### 2.3 Western Blotting

Cells were treated and stimulated as described in 2.1. After 30 min of incubation, Western blot samples were prepared as described before [30].

Western blotting was performed as described previously [24]. Incubation with primary antibodies (mouse anti‐NFAT‐1 (BD Biosciences, Germany), mouse anti‐phospho‐NFAT (R&D Systems, USA), rabbit anti‐β‐actin (Cell Signaling Technology, Germany) was performed overnight at 4°C at a 1:1000 dilution in TBS‐T, containing 5% BSA. Anti‐rabbit or anti‐mouse HRP secondary antibodies (1:2000; GE Healthcare) were used, respectively. For the incubation of the primary antibodies, the membrane was divided at approximately 90 kDa and the upper half of the membrane was incubated in NFAT or phospho‐NFAT, and the lower half of the membrane was incubated in ß‐actin. Immunodetection was performed with Westar Antares reagent (Cyanagen, Italy) using the LAS‐1000 device (Fujifilm Lifescience, Germany). Densitometric quantification was performed with Image J (Rasband, USA). NFAT‐1 and phospho‐NFAT were analyzed at 120 kDa, and β‐actin at 45 kDa.

### 2.4 IL‐2 Quantification

Cells were stimulated as described in 2.1. and harvested after 24 h. Samples were thawed only once for cytokine detection by ELISA. IL‐2 protein concentration was quantified by using OptEIA assays from BD Pharmingen (Heidelberg, Germany) according to the manufacturers' instructions. Samples were measured by using Spark microplate reader (Tecan, Germany).

### 2.5 Intracellular Zinc Measurement

Cells were stimulated as described in 2.1. After 30 min or 3 h, cells were stained in 1 mL measurement buffer for 30 min with 1 µM FluoZin3 AM (Thermo Fisher, Germany) and then processed as described before [26]. Flow cytometry measurements were performed using FACSCalibur Flow Cytometer (BD Biosciences, San Jose, CA, USA). After gating, only structurally normal cells were selected for analyses. For calculation, the formula [Zn] = *K*_D_ × [(*F ‐ Fmin*)/(*Fmax ‐ F*)] was used with the dissociation constant *K*_D_ = 8.9 nM [31]. *Fmin* was determined by the addition of the zinc‐specific, membrane‐permeant chelator TPEN, and *Fmax* was determined by the addition of ZnSO_4_ and the ionophore pyrithione [32].

### 2.6 Intracellular Calcium Measurement

1 × 10^6^ Jurkat cells/mL were stained in PBS with 1 µM Fluo‐4 AM for 30 min at 37°C (Invitrogen by Thermo Fisher Scientific, Eugene, OR, USA). Cells were washed and resuspended in medium to a concentration of 5 × 10^5^ cells/mL. Subsequently, cells were incubated with 50 µM ZnSO_4_ and/or 1 µM pyrithione (Sigma Aldrich) for 15 min or left untreated. Then, cells were centrifuged and resuspended in the same or in fresh medium. Then, cells were stimulated with either 2.5 µg/mL PHA (Biochrom, Germany), 10 ng/mL PMA (Sigma Aldrich) or 1 µM Cal (Sigma Aldrich) for 10 min at 37°C. For calcium measurements, 20 mM EDTA (Fmin; Sigma‐Aldrich) and 2 µM A23187 (Fmax; Tocris, bio‐techne, Minneapolis, MN, USA) were used. Subsequently, the fluorescent intensity was measured using FACSCalibur Flow Cytometer. The mean fluorescent intensity was analyzed with FlowJo Software version 10.8.1 (BD Biosciences). Intracellular calcium concentrations were calculated using the formula [Ca] = *K*_D_ × [(*F ‐ Fmin*)/(*Fmax ‐ F*)] with the dissociation constant *K*_D_ = 335 nM [33].

### 2.7 Statistical Analysis

Data was statistically analyzed using GraphPad Prism 9 software (GraphPad Software Inc., USA). The ROUT method was used to test for outliers, which were removed accordingly. For normality testing the Shapiro–Wilk test was performed. Statistical significances were calculated depending on the normal distribution using either one‐way ANOVA and Tukey's post hoc test, Friedman test and Dunn's post hoc test, or Kruskal–Wallis test and Dunn's post hoc test. The corresponding test and the number of samples are indicated in the figure legends. Statistically significant values do not share the same letters.

## 3 Results

### 3.1 Zinc Inhibits the NFAT Signaling Pathway

Since previous publications showed contradictory effects of zinc on IL‐2 secretion by T cells, we first analyzed the effect of zinc supplementation on the stimulation of Jurkat T cells.

Cells remained untreated or were preincubated with zinc for 15 min. Subsequently, the cells were stimulated with PMA/Cal to induce IL‐2 production. Interestingly, preincubation with zinc led to decreased levels of IL‐2 after stimulation (Figure 1A–C). This effect was significant both after 6 h at mRNA level (Figure 1B) and after 24 h at protein level (Figure 1C). Of note, incubation with zinc alone did not lead to activation of the cells (Figure 2A–C).

We proposed two possible mechanisms for how zinc could impact on the IL‐2 expression. Firstly, the influx of zinc could compete with the influx of calcium and thus decrease the stimulation. Secondly, high intracellular zinc levels could impact directly on signaling molecules and thus modulate activation. To distinguish both effects, the zinc‐supplemented medium was exchanged with zinc‐adequate medium after 15 min of preincubation, indicated as “wash” throughout the manuscript. Interestingly, the inhibiting effect of zinc on both, IL‐2 mRNA expression and protein expression was reversed significantly when supplemented zinc had been washed out (Figure 1A–C). To further characterize the effects of zinc we analyzed the relation of pNFAT to NFAT. Following stimulation, we detected a significantly decreased level of NFAT phosphorylation (Figure 1D,E). Interestingly, this effect is reversed by preincubation with zinc (Figure 1D,E). In line with the IL‐2 production, after removing supplemented zinc from the medium with the washing step, the inhibitory effect of zinc on NFAT dephosphorylation was reduced (Figure 1D,E).

A possible explanation for reduced IL‐2 production under constant zinc supplementation could be that PMA is structurally directly affected by zinc and thus restrained in function. To address this issue, we used PHA as another stimulus of IL‐2 expression. Surprisingly, the inhibiting zinc effect could not be measured (Figure 2A–C). Moreover, zinc‐supplementation even increased the IL‐2 mRNA (Figure 2B). However, this did not translate into a significantly higher protein level (Figure 2C). Of note, the concentration of IL‐2 in the supernatant was only half of what was released following PMA/Cal stimulation.

The calcium influx induced by ionophore Cal during PMA/Cal stimulation may have a pivotal role in these investigations, that is, by competing with zinc influx. For that reason, Cal was added to PHA during Jurkat cell stimulation (Figure 3). As expected, the stimulating effect of PHA on IL‐2 was reinforced by the addition of Cal. However, in Jurkat cells that were preincubated with zinc, this effect was again strongly suppressed (Figure 3A–C). Investigation of the phosphorylation level of NFAT as well revealed a suppressive impact of zinc on Cal‐modulated amplification of PHA stimulation. After removing supplemented zinc from the medium to decrease the extracellular levels of zinc (wash), the suppressing effect disappeared (Figure 3D,E) as it was shown with PMA/Cal (Figure 1).

### 3.2 Pyrithione Increases and Stabilizes Intracellular Zinc Levels

The previous results showed a significant inhibitory effect of zinc on the PMA/Cal‐ or PHA/Cal‐activated NFAT pathway, an effect that had been reversed by washing out extracellular supplemented zinc after 15 min of preincubation. There are two possible explanations why the washing step reversed the zinc effect: The high influx of zinc is essential for the impact on the signaling pathway, as opposed to highly increased intracellular zinc levels during ongoing zinc supplementation that directly interfere with NFAT phosphorylation. The first hypothesis is supported by zinc's suppressing effect appearing only with Cal addition. An increased influx of zinc might be competitive to the Ca^2+^ signal or act as a second messenger in the CN/NFAT pathway. On the other hand, incubation in zinc‐supplemented medium may lead to greatly increased intracellular zinc levels, which could be responsible for the modulation of the signaling pathway. Thus, we analyzed the intracellular zinc concentration and found that following all stimulations in the presence of zinc there was a significant increase in intracellular zinc after 3 h. However, we found no increase in the samples where extracellular zinc was removed after preincubation (Figure 4A–D). To be able to distinguish between the two possibilities of zinc‐mediated IL‐2 suppression discussed above we aimed to reach significantly elevated intracellular zinc levels already after 15‐min preincubation to investigate the impact of intracellular zinc amount independently from increased extracellular influx during ongoing zinc supplementation.

In our opinion the zinc ionophore pyrithione seemed like a helpful tool in this experiment as it was previously described to boost intracellular zinc levels within a short time [34]. Pyrithione binds to zinc and enters the cell via membrane transport to passive diffusion [35]. It was reported to be selective to the divalent metal‐ions zinc and copper [36]. For that reason, we do not expect a competitive intake of Ca^2+^. When Jurkat cells were preincubated with zinc/pyrithione for 15 min before medium was exchanged with zinc‐adequate, pyrithione‐free medium we found that the intracellular zinc level remained significantly increased after 3 h compared to unstimulated control cells, and reached zinc levels that were statistically similar to those cells with a constant supply of extracellular zinc (Figure 4A). Crucially, zinc levels of zinc‐supplemented cells and cells shortly boostered with zinc/pyrithione were comparable in all stimulation conditions (Figure 4B–D). We assumed that our experimental design to investigate the impact of increased intracellular zinc levels independently from extracellular influx by using zinc/pyrithione as a booster can be used reliably.

### 3.3 Intracellular Zinc Causes Inhibition of IL‐2 Expression in Jurkat T Cells

After setting up the protocol to booster intracellular zinc during preincubation, we measured IL‐2 production by Jurkat cells on mRNA levels after 3 and 6 h and on protein level after 24 h of PMA/Cal (Figure 5) or PHA (Figure 6). When Cal was combined with zinc alone, no IL‐2 production and secretion could be measured (Figure 6B,C). When PMA or PHA were combined with the ionophore Cal, zinc supplementation led to a significant decrease in IL‐2 production (Figures 5B,C and 6B,C). Interestingly, this effect was consistently detected in both, cells with constant supply of extracellular zinc and cells in which zinc was only increased intracellularly by preincubation with zinc/pyrithione (Figures 5B,C, and 6B,C). Thus, we showed that the increase of intracellular zinc rather than increased zinc influx possibly competing with calcium is responsible for the diminishing effect on IL‐2 expression.

### 3.4 Zinc Does Not Have a Significant Impact on Calcimycin‐Mediated Calcium Influx

A possible explanation for the previous results is that zinc could interfere with the influx of calcium. If zinc reduces the entry of calcium to the cell by inhibiting calcium transporters, lower intracellular calcium levels would result in lower IL‐2 expression. To test this hypothesis, we measured the intracellular calcium level using the experimental setup as described above. When PHA was combined with Cal, there was a significant increase in intracellular calcium level. Interestingly, there was no effect on calcium level, when zinc was added (Figure 7A). There was no difference when pyrithione was used as an intracellular zinc booster. The effects are similar with PMA/Cal as a stimulus, even though in this experiment the calcium level was not significantly increased by stimulation with Cal (Figure 7B).

## 4 Discussion

The major impact of zinc on signaling pathways was established decades ago and is subject of current research because the molecular mechanisms are still not fully revealed.

It was previously described that zinc exhibits an inhibitory effect on several signaling pathways of the human immune system [37, 38, 39]. However, depending on context, zinc also has an activating effect on multiple signaling processes [40, 41]. For example, it was shown that zinc inhibits phosphatase activity, which leads to phosphorylated and activated signaling molecules in the ERK pathway [42].

Zinc inhibits the phosphatase CN and modulates the CaMPK‐II [43, 44]. Many signaling pathways that are being regulated by calmodulin are also affected by zinc [45]. Over the decades, contradictory findings regarding the influence of zinc on IL‐2 expression have been made. Our study aimed to elucidate the impact of zinc supplementation on the IL‐2 production in Jurkat cells. A previous study published by Tanaka et al. discovered that zinc inhibits the NFAT induced IL‐2 expression in Jurkat T cells [27]. This was confirmed in our first experiments at phosphorylation level, at mRNA level, and by measuring the secreted IL‐2 concentration following PMA/Cal stimulation. The relative expression of IL‐2 mRNA in Jurkat cells that were only stimulated by PHA was much lower compared to stimulations including Cal, indicating a low level of activation. Interestingly, zinc had no effect on IL‐2 production, when only PHA was used as stimulus, suggesting that an involvement of Ca^2+^ signaling is mandatory to observe the inhibitory effect of zinc. When extracellular zinc was washed out after 15 min, no inhibition of zinc on IL‐2 signaling was measured. This could be explained through previous findings by Hershfinkel et al., who established the role of selective G‐protein coupled receptor ZnR/GPR39, activated by transient increase in extraceullar zinc [46]. It mediates calcium release from the ER leading to enhanced activation of calcium‐dependent signaling [47].

As described before, stimulation with Cal induces a calcium influx [48]. We found that zinc diminished calcium's activating effect facilitated by Cal [49]. Two possible mechanisms can underlie this phenomenon: (1) Increased zinc influx during zinc supplementation might compete with the Ca^2+^ signal and lead to an inhibition or (2) an intracellular increase of zinc results in suppressed cell activation, possibly by direct modulation of phosphatase activity.

Treatment with zinc/pyrithione creates a strong intracellular zinc intake [50]. In our work, we showed that a 15‐min preincubation with zinc/pyrithione led to high intracellular zinc levels comparable to a constant zinc supplementation in the medium and stable over several hours. Our experiments showed further that this increased intracellular zinc levels suppressed IL‐2 expression as effectively as a constant zinc supply. This led to the suggestion that increased zinc in the cytoplasm inhibits IL‐2 expression, regardless of the presence of excess extracellular zinc and would confirm the second hypothesis that zinc modulates phosphatase activity directly. Yu et al. demonstrated that T cell activation leads to a rapid zinc influx within seconds [51]. According to the authors, the increase was dependent on extracellular zinc concentrations. The influx was essential for a decreased activation of the phosphatase SHP‐1, an established molecule which acts as a negative feedback regulator to the T cell signaling [52, 53]. By inhibiting negative feedback to the TCR signaling, zinc mediated a strong calcium influx after stimulation. It was previously reported that zinc deficiency led to decreased expression of IL‐2 caused by the reduced activating effect of zinc on NFκB pathway [25]. Daaboul et al. investigated that zinc supplementation activates signaling molecules of the NFκB pathway, which resulted in an increased IL‐2 production [26]. Our results show that the supplementation of Jurkat cells with higher concentrations of zinc in the medium during preincubation leads to a significant decrease of IL‐2 expression and an increased proportion of pNFAT. On the other hand, Yamasaki et al. discovered a rapid zinc release from the ER dependent on calcium influx and activation of the MAPK/ERK pathway, a phenomenon they called zinc wave [54]. They showed that the zinc wave came from intracellular sources and acted as a second messenger by enhancing NFκB DNA‐binding activity [55]. The results of our study with IL‐2 production in Jurkat cells would be in line with their observations, however, they described the zinc wave only in mast cells, not in T cells. Previous findings further suggest that phorbol ester treatment results in an increase of intracellular zinc caused by redistribution from cell organelles [56]. However, when we stimulated our cells with PMA/Cal or PHA/Cal we discovered a slight but not significant increase of free zinc compared to the control cells. This fact was described before by Haase et al., who demonstrated a PMA‐induced zinc release which occurred in monocytes but not in lymphocytes [32]. Interestingly, no augmented zinc amount after stimulation was detectable when Cal was not involved, which is in line with our findings showing even a slight decrease in intracellular zinc after PHA stimulation.

**Figure 8:**
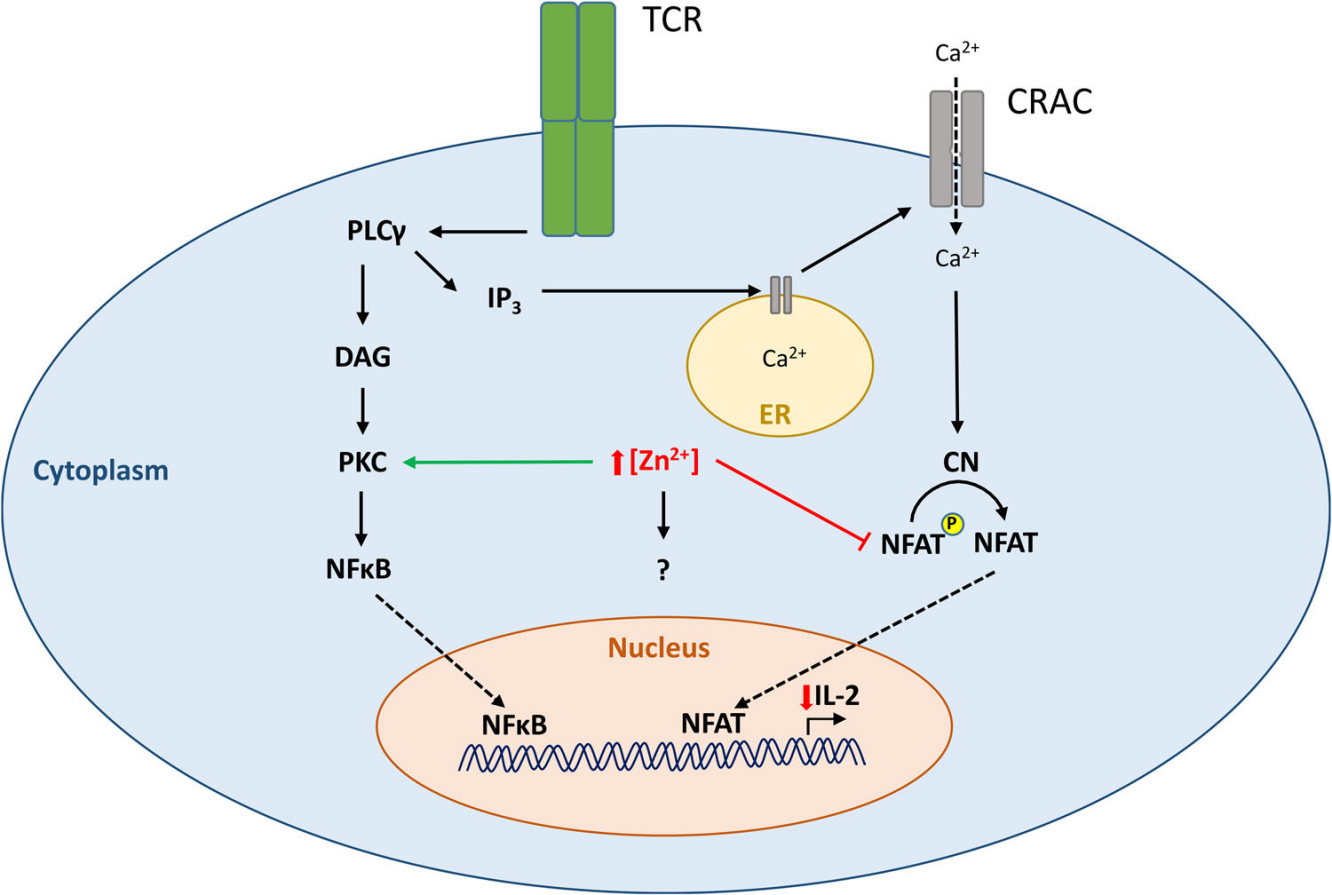
The impact of increased intracellular zinc on signaling pathways in T cells. Schematic view of the effects of zinc on IL‐2 related pathways. The inhibitory effect of zinc on pNFAT is represented by the red bar. Solid‐lined arrows indicate an activating impact. Dotted arrows indicate translocation of calcium or signaling molecules within the cell. The curved arrow indicates dephosphorylation of pNFAT. Upward arrow indicates an increased intracellular concentration of Zn^2+^, with a concurrent decrease in IL‐2 production, indicated by the downward arrow. The question mark indicates a possible additional signaling molecule which would be affected by zinc.

Taken together, our data suggest a major influence of intracellular zinc on Ca^2+^‐dependent signaling pathways. In a schematic overview we summarize what has been found in the literature and what has been detected by our experiments (Figure 8). Prior working groups proposed an activating impact of zinc on PKC signaling [3, 45]. We investigated that zinc had a suppressing effect on Ca^2+^‐dependent IL‐2 expression, which was detectable by a decreased dephosphorylation of pNFAT following stimulations involving Cal. It has been shown that zinc has the potential to change the conformation of CaMPK‐II, which leads to an inability to bind Ca^2+^/Calmodulin to activate the CN pathway [44]. Our findings support previous reports that intracellular zinc binds at different sites of the same protein, instead of competing with Ca^2+^ for the same binding site [57, 58]. It leads to an inhibiting effect of the Ca^2+^‐depended activation and a significant decrease in IL‐2 production. In contrast, prior working groups proposed an activating impact of zinc on PKC signaling [59]. These contradictory findings raise the question if zinc acts as a second messenger through a still unknown independent pathway.

Even though we revealed that the intracellular zinc elevation is crucial for inhibition IL‐2 expression, the cellular membrane transport cannot be excluded. It has been shown that some types of Cal are also selective for other divalent cations, such as Zn^2+^ [60] which might lead to an influx of zinc into cytoplasm during the stimulation and increase the disequilibrium between Ca^2+^ and Zn^2+^, facilitating the inhibitory effect of Zn^2+^. However, the influx of zinc via Cal should be the same in control and zinc‐boostered cells and thus does not explain the inhibitory effect. Over the decades, numerous working groups reported that zinc has an inhibiting effect on Ca^2+^ channels [28, 61]. As we already know, zinc can block ion channels, therefore leading to a lower calcium level intracellularly. Hence, we needed to consider zinc blocking the ion channels to have approximately the same effect as inhibiting the signaling pathway directly. However, the results of our experimental setup showed no impact of zinc on the intracellular calcium level in cells stimulated in combination with Cal. If zinc interacted with calcium, or acted competitively, or directly blocked the channel in our setting, a decrease in calcium levels would be observed. The results obtained also support the hypothesis that zinc uses its own previously unknown pathway. Another difficulty lies in various effects of the stimulators on signaling pathways and their cross‐linking to each other. PMA/Cal is known to stimulate PKC, which activates mainly the NFκB pathway [17]. Our experiments also clearly demonstrate the effect of PMA/Cal on NFAT as previously shown by others [62, 63]. To separate the individual signaling pathways more clearly, the effect of zinc in combination with selective inhibitors should be examined in future studies. Additionally, a limitation of this study is that the experiments were conducted with Jurkat T cells. Future studies need to show whether the same findings can be reproduced in primary cells for example by experiments with human separated T cells from healthy blood donors.

IL‐2 is essential for the maintenance of Treg cells and the differentiation of CD4^+^ cells in multiple T cell subsets [64]. A dysfunction of IL‐2 and the IL‐2 receptor can lead to excessive immune responses, which may result in numerous autoimmune diseases, like type 1 diabetes, multiple sclerosis, and rheumatoid arthritis [65]. It could be a helpful tool to use zinc's immunomodulatory capacity to inhibit dysregulated cytokines like IL‐2 and to prevent autoimmune diseases. In conclusion, our study has contributed to a better understanding of the effect of zinc on IL‐2 signaling in T cells. Due to our research results, it may be possible in the future to expand the therapeutic field of immunosuppressants through the systemic use of zinc.

## Author Contributions

H.J.F. and L.R. designed the study. C.M.S. performed the experiments. J.J. measured the intracellular calcium concentration. C.M.S. analyzed the data. J.J. data curation. C.M.S. wrote and H.J.F., L.R., and J.J. revised the manuscript.

## Conflicts of Interest

The authors declare no conflicts of interest.

## Data Availability

The data that support the findings of this study are available from the corresponding author upon reasonable request.
